# The Book World of Medicine and Science

**Published:** 1904-02-13

**Authors:** 


### THE BOOK WORLD OF MEDICINE AND SCIENCE.

A SCHEME OF BRAIN STORAGE. By FREDERICK R. FARMER, M.R.C.S., L.R.C.P., formerly Assistant Medical Officer, Fisherton House, Salisbury, and Wells County Asylum. (London: Kegan Paul, Trench, Trübner and Co. 1903.)

DR. FARMER'S book on the subject of brain storage contains novel ideas, ambiguously expressed, as to the influence of environment on the structure of the human brain. He seeks to explain the method whereby impressions are stored, but unfortunately his manner of expression is lacking in scientific precision, and the presentation of his material is so disjointed that it is by no means easy to follow his would-be argument. He would appear to hold that storage of impressions takes place in the polymorphous cells underlying the pyramids, that each cell contains the impressions received in a definite period of time, and that, as time elapses, these cells sink more deeply into the cortical layers, being superseded by other fresh ones. We fear the various views expressed in this pamphlet will not further us in the understanding of memory, or afford any directing light on the problems connected with a study of the influence of environment on mental development.

THE PREVENTION OF CONSUMPTION. By ALFRED HILLIER, M.D., C.M., B.A., Secretary to the National Association for the Prevention of Consumption, etc. Revised by Professor R. KOCH. Pp. 226. With illustrations. (London: Longmans, Green and Co. 1903. Price 5s.)

THIS well-intentioned manual is disappointing. The author has rightly perceived that there is need for a well-balanced and judiciously expressed pronouncement on the tuberculosis question viewed in its entirety and as a great social problem. The attempt to meet this certainly existing want has failed mainly in consequence of a desire to push, what is suggested as progress, far beyond the firm foundations accorded by pathology. Dr. Hillier would appear to pose rather as a pioneer than a pathologist, and in his enthusiasm for coercive measures is apt to fall into the phraseology and methods of the dogmatic teacher. This book is apparently intended for the public rather than designed for the strictly medical reader, and hence we think particular caution should have been taken to avoid anything likely to pander to that morbid condition of mind which has been conveniently designated phthisophobia. The work is incomplete and in parts is best described as scrappy. The opening chapter on the history and nature of tuberculosis is altogether inadequate. More than a page is devoted to a discussion of the "influence of tuberculosis on art," and much space is given to Koch's discovery of the tubercle bacillus, but practically nothing appears respecting much of such recent work as is going far to modify our views as to the infectivity of the disease. There is a slipshod laxity in expression which is much to be deplored; the following is only one of many examples:—"In the case of tuberculosis, in all its forms, the most important mode of entrance is that by inhalation into the respiratory tract." We think neither grammarians nor pathologists will applaud this manner of dogmatic statement. Dr. Hillier is a great believer in the infectivity of phthisis. He also seems to consider Flügge's cough spray a factor of much importance in its spread. Compulsory notification is strongly advocated. A chapter is devoted to a consideration of the relation of bovine to human tuberculosis and while Koch's views receive careful attention the investigations of many other well-known pathologists are not even mentioned. In the section on personal precautions there is much sound advice, but even here the matter is dealt with in a too superficial fashion. But perhaps the most disappointing section is that which deals with sanatoria and institutions for the consumptive of all classes and conditions. Dr. Hillier, it is true, describes the particular sanatorium with which he is connected, but he does not furnish any reliable guide to the many excellent private and public establishments which are now accomplishing admirable work in this country. His attempt to deal with the factors which go to constitute sanatorium treatment is almost ludicrous in its meagreness: only 21 lines are devoted to a consideration of the influence of wind and less than two and a half lines completes the reference to sunshine and humidity. Dr. Hillier would seem to be blinded by German glamour and gives much space to a consideration of the methods of the German Imperial Insurance Department which of course is not in any way applicable in this country. The most useful portion of the book is the Appendix, which contains much material likely to be of real service.

THE STERILISATION OF URETHRAL INSTRUMENTS. By HERBERT T. HERRING. (H. K. Lewis. Pp. 176. Illustrated. Price 5s.)

THE author bemoans the fact that so little attention is given to the ordinary rules of surgical cleanliness in urinary surgery, and the object of the book is to combat this tendency by presenting, in a form comprehensible to the general public, the rules that should guide either the practitioner or the patient in performing any act of instrumentation. In the opening chapters a brief popular description is given of the anatomy of the urinary passages, and is followed by an account of the principal instruments, bougies, catheters, etc., which are in common use. Several succeeding chapters are then devoted to various methods of sterilising instruments, some of them simple and capable of being carried out by anyone of average intelligence, and others of a more complicated nature. By these methods the author suggests that absolute sterility may be obtained and the passage of an instrument be rendered almost, if not quite, free of any danger of infecting the urinary passages from without. There is one criticism we feel called on to make, namely, the lack of reference made to the possibility of infection of instruments by means of organisms living in the urethra. That organisms do infest the normal urethra has been proved by many observers, and it has been found that to obtain absolute sterility it is necessary before passing any instrument to flush out the urethra. The subsequent chapters of the book are devoted to giving a rather popular description of various urinary complaints with methods of treatment. On the advisability of this procedure there may be some difference of opinion, but as regards the preliminary part of the book there can be no doubt that if patients can be prevailed upon to take precautions as to the cleanliness of their instruments, a great step will have been gained, and the author will have performed a good work in setting the various details by which this may be obtained clearly and concisely before them.

